# Plate-to-Layer Bi_2_MoO_6_/MXene-Heterostructured Anode for Lithium-Ion Batteries

**DOI:** 10.1007/s40820-019-0312-y

**Published:** 2019-09-25

**Authors:** Peng Zhang, Danjun Wang, Qizhen Zhu, Ning Sun, Feng Fu, Bin Xu

**Affiliations:** 1 State Key Laboratory of Organic-Inorganic Composites, Beijing Key Laboratory of Electrochemical Process and Technology for Materials, Beijing University of Chemical Technology, Beijing, 100029 People’s Republic of China; 2 Shaanxi Key Laboratory of Chemical Reaction Engineering, School of Chemistry and Chemical Engineering, Yan’an University, Yan’an, 716000 People’s Republic of China

**Keywords:** Bi_2_MoO_6_, MXene, Electrostatic self-assembly, Heterostructure, Lithium-ion batteries

## Abstract

Bi_2_MoO_6_ is a potentially promising anode material for lithium-ion batteries (LIBs) on account of its high theoretical capacity coupled with low desertion potential. Due to low conductivity and large volume expansion/contraction during charge/discharge cycling of Bi_2_MoO_6_, effective modification is indispensable to address these issues. In this study, a plate-to-layer Bi_2_MoO_6_/Ti_3_C_2_T_*x*_ (MXene) heterostructure is proposed by electrostatic assembling positive-charged Bi_2_MoO_6_ nanoplates on negative-charged MXene nanosheets. MXene nanosheets in the heterostructure act as a highly conductive substrate to load and anchor the Bi_2_MoO_6_ nanoplates, so as to improve electronic conductivity and structural stability. When the mass ratio of MXene is optimized to 30%, the Bi_2_MoO_6_/MXene heterostructure exhibits high specific capacities of 692 mAh g^−1^ at 100 mA g^−1^ after 200 cycles and 545.1 mAh g^−1^ with 99.6% coulombic efficiency at 1 A g^−1^ after 1000 cycles. The results provide not only a high-performance lithium storage material, but also an effective strategy that could address the intrinsic issues of various transition metal oxides by anchoring them on MXene nanosheets to form heterostructures and use as anode materials for LIBs.

**Electronic supplementary material:**

The online version of this article (10.1007/s40820-019-0312-y) contains supplementary material, which is available to authorized users.

## Highlights

- The highly conductive Ti_3_C_2_T_*x*_ (MXene) is introduced as a substrate for loading Bi_2_MoO_6_.
- The Bi_2_MoO_6_/MXene heterostructure exhibits ultra-long cycle durability and superior rate capability.
- Electrochemical kinetic mechanism is analyzed for the as-prepared heterostructure.

**Electronic supplementary material:**

The online version of this article (10.1007/s40820-019-0312-y) contains supplementary material, which is available to authorized users.

## Graphical abstract

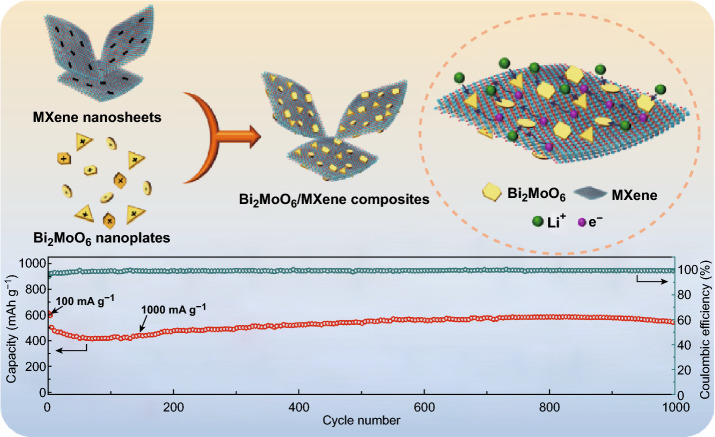

## Introduction

Lithium-ion batteries (LIBs), with advantages of high energy density, environmental benignity, and no memory effect, have occupied unparalleled markets of electric vehicles and portable electronics [1–3]. However, the low theoretical capacity (372 mAh g^−1^) of the commercial graphite anode material actually limits the further development of LIBs due to the booming demand for higher energy density in applications, which urges researchers to explore high-performance anode materials for next-generation LIBs [4, 5]. On this account, a variety of transition metal oxides (TMOs) have been investigated as anode materials for LIBs because of their high theoretical capacity and moderate cost [6–8], e.g., SnO_2_ [9, 10], Fe_3_O_4_ [11], and Mn_3_O_4_ [12]. Nevertheless, owing to their alloying or conversion storage mechanism, the huge volume expansion/contraction of TMOs during lithiation/delithiation processes results in the instability of the electrode structure and the repeated cracking/forming of the solid electrolyte interface (SEI), leading to the capacity fading together with continuous consumption of electrolyte [13–16]. For example, SnO_2_ can store up to 4.4 units of Li in one unit of Sn, endowing with a high theoretical capacity of 790 mAh g^−1^ as well as a volume charge of more than 200% [17]. Decreasing the particle size of TMOs to nanoscale coupled with morphological control (e.g., nanotubes [18], nanospheres [19], and nano-flowers [20]) provides a promising direction to improve the cycling stability of TMOs [16, 21, 22]. In addition, the intrinsic low electrical and ionic conductivity renders the TMOs with unacceptable rate capability, which also obstructs its application as electrode material of LIBs [6, 16, 21]. An efficient strategy to overcome this issue is to rationally design composites combining TMOs and highly conductive materials, such as graphene [23], carbon nanotubes [24], and amorphous carbon [25].

Among various TMOs, bismuth molybdate (Bi_2_MoO_6_) with a structure of alternate [Bi_2_O_2_]^2+^ layers and [MoO_4_]^2−^ perovskite layers can be potentially used as an anode material for LIBs due to its high theoretical capacity (791 mAh g^−1^) as well as low desertion potential (< 1.0 V) [26, 27]. Up to date, most reports about Bi_2_MoO_6_ focused on its photocatalyst properties, while its application in LIBs, which has great significance, still needs further development. When used as anode material, the main restricts of Bi_2_MoO_6_ are similar to those of other TMOs, i.e., the large volume change during lithiation/delithiation and the intrinsic low conductivity. Thus, the strategies of rational design on the structure and combination with conductive materials can also be employed for improving electrochemical performances of Bi_2_MoO_6_ electrode material [27–31]. For example, Zhai et al. [28] reported a Bi_2_MoO_6_/reduced graphene oxide (rGO) composite based on the in situ growth of Bi_2_MoO_6_ on the rGO substrate, in which the rGO provides high conductivity and is beneficial for exposing the active sites and alleviating the volume change of Bi_2_MoO_6_.

More recently, two-dimensional (2D) transition metal carbides and nitrides known as MXenes have been widely studied because of their high metallic conductivity, tailorable surface chemistries, and mechanical flexibility [32–35]. Typically, MXenes are synthesized by selectively etching the A layer (group IIIA or group IVA element) from the ternary precursors known as MAX phases and have a general formula of M_*n+1*_X_*n*_T_*x*_, where M is an early transition metal; X stands for C and/or N; *n* = 1, 2, or 3; T_*x*_ represents the surface functional groups, such as –O, –F, and –OH [36, 37]. According to the calculation result, Ti_3_C_2_T_*x*_, one of the most studied MXenes possesses lower lithium diffusion barrier (~ 0.07 eV) compared with that of the graphite carbons (~ 0.3 eV), indicating faster Li^+^ transport and higher lithiation/delithiation rate than the commercial graphite anode [38, 39]. In this regard, Ti_3_C_2_T_*x*_ MXene could be an ideal substrate to combine with various TMOs (e.g., SnO_2_ [40, 41], Fe_3_O_4_ [42], Sb_2_O_3_ [43], LiMn_2_O_4_ [44]) to fabricate high-performance electrode materials for LIBs. For example, Zhao et al. [45] fabricated a flexible Ti_3_C_2_T_*x*_/NiCo_2_O_4_ hybrid film via an in situ growth method. As the Ti_3_C_2_T_*x*_ facilitated fast ion transport and electron transfer, the electrochemical performance of the film electrode was effectively enhanced. Therefore, integrating with the highly conductive Ti_3_C_2_T_*x*_ MXene is expected to significantly improve the lithium storage performances of Bi_2_MoO_6_.

In this work, for the first time, Bi_2_MoO_6_/MXene (Ti_3_C_2_T_*x*_) composites with a plate-to-layer heterostructure have been fabricated through a simple electrostatic self-assembly followed by freeze-drying method. Based on the electrostatic interaction, the positive-charged Bi_2_MoO_6_ nanoplates are uniformly anchored on the surface of the negative-charged MXene nanosheets, leading to a synergistic effect between the Bi_2_MoO_6_ nanoplates and the MXene nanosheets with several merits: (i) The Bi_2_MoO_6_ nanoplates distributed on the MXene can sufficiently expose the active sites for high capacity and simultaneously serve as spacers to prevent the MXene nanosheets from restacking, so could greatly enhance the ion accessibility. (ii) The MXene can effectively prevent the Bi_2_MoO_6_ nanoplates from aggregation and alleviate their huge volume change in lithiation/delithiation processes, effectively avoiding the loss of the active sites and ensuring the cycle stability. (iii) The MXene nanosheets contact closely with the Bi_2_MoO_6_ nanoplates, which can enhance the electronic conductivity and facilitate Li^+^ diffusion, in favor of the rate performance. Consequently, acting as an anode material for LIBs, the Bi_2_MoO_6_/MXene heterostructures exhibit high capacity, superior rate capability, and excellent cycling stability, demonstrating not only a promising LIBs anode material, but also the effectiveness of MXene substrate in place to enhance the electrochemical performance of TMOs.

## Experimental

### Materials Syntheses

#### Synthesis of Ti_3_C_2_T_*x*_ MXene

The MXene nanosheets were obtained as reported previously [40, 46, 47]. Typically, 0.99 g of lithium fluoride (LiF) was added to 10 mL of 12 M hydrochloric acid (HCl) under stirring for dissolving. Then, 1 g of Ti_3_AlC_2_ powder (400 mesh, purchased from 11 Technology Co. Ltd) was slowly added to the above solution. The mixture was stirred at 35 °C for 24 h to ensure the complete etching. After that, the residue was washed with deionized water for several times until the pH of the supernatant is above 6. The precipitate was then re-dispersed in deionized water followed by sonication for 1 h under Ar atmosphere. After centrifugation at 5200 rpm for 1 h, the supernatant was collected as the MXene aqueous solution. In order to determine the concentration of the MXene aqueous solution, 5 mL of MXene aqueous solution was filtered and vacuum dried to obtain a pristine MXene film. After weighting the film, the concentration was obtained. The concentration of MXene aqueous solution was diluted to 2 mg mL^−1^.

#### Synthesis of the Bi_2_MoO_6_ Nanoplates

The Bi_2_MoO_6_ nanoplates were synthesized as reported previously [48]. Typically, 2 mmol of Bi(NO_3_)_3_·5H_2_O was added to 10 mL of HNO_3_, while 0.143 mmol of (NH_4_)_6_Mo_7_O_24_·4H_2_O was dissolved in 10 mL of deionized water, respectively. After stirring for 30 min, the Bi(NO_3_)_3_·5H_2_O solution was added to the (NH_4_)_6_Mo_7_O_24_·4H_2_O solution dropwise under stirring for 60 min. Once approached to pH neutral (adjusted by ammonium hydroxide), the mixture was transferred into a Teflon-lined stainless steel autoclave and then heating at 180 °C for 12 h. Subsequently, the product was washed with deionized water and absolute ethanol for several times and dried under vacuum to obtain the pristine Bi_2_MoO_6_ nanoplates.

#### Synthesis of the Bi_2_MoO_6_/MXene Heterostructures

The composite Bi_2_MoO_6_/MXene heterostructures were prepared via an electrostatic self-assembly method. The positive-charged Bi_2_MoO_6_ nanoplates was added to deionized water and sonicated for 30 min to form the Bi_2_MoO_6_ suspension (1 mg mL^−1^). Then, the Bi_2_MoO_6_ suspension was mixed with the MXene solution (1 mg mL^−1^) under continuous stirring for 30 min with various ratios. The mixture was freeze-dried for 48 h to obtain the Bi_2_MoO_6_/MXene heterostructures (denoted as Bi_2_MoO_6_/MXene-x, where x stands for the mass ratio of MXene in the heterostructures).

### Materials Characterization

The morphology of the prepared Bi_2_MoO_6_, MXene nanosheets, and Bi_2_MoO_6_/MXene heterostructures was observed through scanning electron microscope (SEM, Hitachi S4800), transmission electron microscope (TEM, Hitachi HT7700), and scanning transmission electron microscopy equipped with energy-dispersive X-ray spectroscopy (EDS) for elemental mapping (STEM, Hitachi HT7700). Zeta potentials were measured by a Marlvern laser particle size analyzer (ZS980). XRD patterns were performed using X’Pert-Pro MPD (PANalytical, the Netherlands) diffractometer with monochromatic Cu Ka radiation (*λ* = 1.5418 Å, with scan speed of 4° min^−1^). Raman spectra were conducted through Raman spectrometer (Renishaw 1000) with a 1 mW He–Ne laser (633 nm) as an irradiation source. XPS analysis was performed by ESCALAB 250 (ThermoFisher Scientific, USA). AFM image was collected using atomic force microscope (Dimension ICON).

### Electrochemical Measurements

All the electrochemical measurements were taken by assembling CR2025 coin-type cells in Ar-filled glove box at room temperature. The working electrodes were fabricated by mixing active materials (Bi_2_MoO_6_/MXene heterostructures or Bi_2_MoO_6_) conductive agent (Super-P) and binder (carboxymethylcellulose sodium, CMC) in deionized water with a mass ratio of 70:20:10 followed by coating the mixed slurry onto copper foil. The mass loading of the active materials on the current collector was fixed to 0.8 mg cm^−2^ in order to standardize the test results. Lithium foil, Celgard 3500 membrane, and 1 M LiPF_6_ in ethylene carbonate (EC)/diethyl carbonate (DEC) (1:1 by volume) with an addition of 5 wt% fluoroethylene carbonate (FEC) were employed as counter electrode, separator, and electrolyte for LIBs, respectively. The amount of electrolyte used in each coin cell is 120 μL. The galvanostatic charge/discharge tests were carried out on Land BT2000 battery tester (Wuhan, China) in the voltage range of 0.01–3 V. Cyclic voltammetry (CV) measurements were taken on the VSP electrochemical workstation (Bio-Logic, France) with a potential window of 0.01–3 V at a scan rate of 0.1 mV s^−1^. Electrochemical impedance spectroscopy (EIS) was tested in the frequency range of 100 kHz to 0.1 Hz with amplitude of 10 mV. The galvanostatic intermittent titration technique (GITT) was carried out with current pulse (100 mA g^−1^ for LIBs) for 30 min followed by 1 h relaxation on a Land BT2000 battery tester (Wuhan, China). The diffusion coefficients were calculated from the GITT potential profiles according to the Fick’s second law with the following Eq. 1:

1 $$D = \frac{4}{\pi \tau }\left( {\frac{{m_{B} V_{M} }}{{M_{B} S}}} \right)^{2} \left( {\frac{{\Delta E_{S} }}{{\Delta E_{\tau } }}} \right)^{2}$$

where $\tau$ stands for the duration of current pulse; *m*_*B*_ represents the mass of active material in the pole; *V*_*M*_ and *M*_*B*_ is the molar volume and the molar mass of the active materials, respectively; *S* is the geometric area of the electrode; $\Delta E_{\tau }$ means the potential variation during the current pulse; and $\Delta E_{S}$ represents the quasi-thermodynamic equilibrium potential variation before and after the current pulse.

## Results and Discussion

**Fig. 1:**
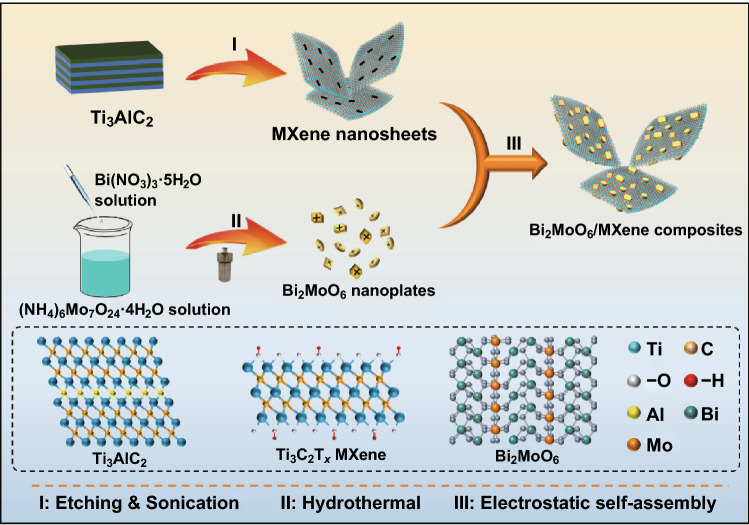
Schematic diagram for the simple electrostatic self-assembly of positive-charged Bi_2_MoO_6_ nanoplates on the negative-charged MXene nanosheets

**Fig. 2:**
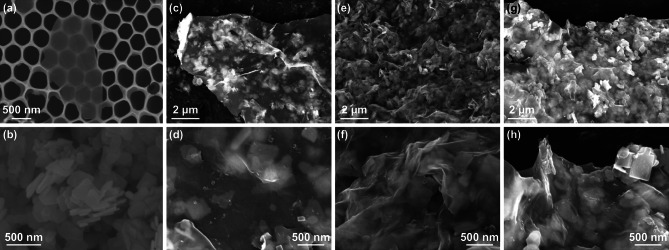
SEM images of **a** MXene nanosheets, **b** Bi_2_MoO_6_ nanoplates, **c**, **d** Bi_2_MoO_6_/MXene-50%, **e**, **f** Bi_2_MoO_6_/MXene-30%, and **g**, **h** Bi_2_MoO_6_/MXene-10%

Figure 1 illustrates the synthetic process for the Bi_2_MoO_6_/MXene heterostructure. First, the delaminated Ti_3_C_2_T_*x*_ MXene nanosheets were produced by etching aluminum (Al) layers from Ti_3_AlC_2_ precursor with a solution of LiF and HCl, followed by sonication and the subsequent centrifugation. As shown in Fig. S1, the SEM image indicates the Ti_3_AlC_2_ MAX phase has a layered structure, and the XRD pattern is consistent with that of the previous reports [32, 33]. Single or few-layered MXene nanosheets can be readily obtained through such a mild delamination route with LiF/HCl, which is conducive to loading the Bi_2_MoO_6_ nanoplates. The prepared MXene has a lateral size of 1–3 μm (Fig. 2a) with a thickness below 1.83 nm, indicating 1 or 2 layers on a substrate (Fig. S2). The Bi_2_MoO_6_ nanoplates were synthesized from Bi(NO_3_)_3_·5H_2_O and (NH_4_)_6_Mo_7_O_24_·4H_2_O via a hydrothermal method, leading to the presence of amino group on the surface of Bi_2_MoO_6_ nanoplates and thus positively charged. The synthesized Bi_2_MoO_6_ displays irregular plate-like morphology (Figs. 2b, S3a, b) with a lattice spacing of 0.277 nm, corresponding to the (200) plane of orthorhombic Bi_2_MoO_6_ (Fig. S3c) [31]. Its structure was further confirmed by the EDS mappings, which show a rough proportion of Bi, Mo, and O elements, coupled with the uniform distribution (Fig. S4). The Bi_2_MoO_6_ crystal structure intrinsically consisting of alternate [Bi_2_O_2_]^2+^ layers and [MoO_4_]^2−^ perovskite layers offers open and stable channels for Li^+^ insertion/extraction, endowing Bi_2_MoO_6_ with greatly improved rate capability compared with other TMOs. However, the intrinsic low conductivity and the large volume change during lithiation/delithiation of Bi_2_MoO_6_ still require the modification with conductive materials [28, 30]. The assembly of the Bi_2_MoO_6_ nanoplates with the Ti_3_C_2_T_*x*_ MXene nanosheets was achieved by adding the Bi_2_MoO_6_ dispersion into the MXene solution and mixing thoroughly under continuous stirring. In order to confirm the successful electrostatic self-assembly of the Bi_2_MoO_6_/MXene heterostructure, the zeta potentials of the as-prepared heterostructures coupled with the two components, i.e., the pristine Ti_3_C_2_T_*x*_ nanosheets and the Bi_2_MoO_6_ nanoplates, were measured. As shown in Fig. S5, the as-prepared MXene nanosheets are negatively charged with a zeta potential of − 40.5 mV, which is ascribed to the presence of surface functional groups (e.g., –O, –F, and –OH) [32, 33]. When the positive-charged Bi_2_MoO_6_ (10.1 mV) mixed with the negative-charged MXene, Bi_2_MoO_6_ nanoplates were anchored on the MXene nanosheets based on an electrostatic interaction. Therefore, the zeta potentials of the Bi_2_MoO_6_/MXene heterostructures shift down with the increase in the MXene content, demonstrating the effective electrostatic process. In the assembled heterostructure, the highly conductive MXene nanosheets are expected to significantly enhance the conductivity and alleviate the volume expansion/contraction of Bi_2_MoO_6_ during lithiation/delithiation, thus improving the rate performance and cycling stability.

**Fig. 3:**
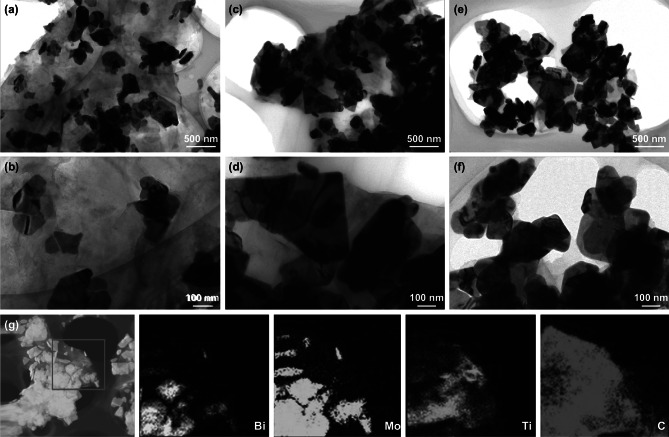
TEM images of **a**, **b** Bi_2_MoO_6_/MXene-50%, **c**, **d** Bi_2_MoO_6_/MXene-30%, and **e**, **f** Bi_2_MoO_6_/MXene-10%. **g** STEM and corresponding element (Bi, Mo, Ti, and C) mapping images of the Bi_2_MoO_6_/MXene-30%

The morphologies of the Bi_2_MoO_6_/MXene heterostructures are shown in Fig. 2c–h. The composite samples were named with the mass ratio of Bi_2_MoO_6_/MXene: 50:50 (Bi_2_MoO_6_/MXene-50%, Fig. 2c, d), 70:30 (Bi_2_MoO_6_/MXene-30%, Fig. 2e, f), and 90:10 (Bi_2_MoO_6_/MXene-10%, Fig. 2g, h). It is apparent that with increasing Bi_2_MoO_6_ content, more Bi_2_MoO_6_ nanoplates cover on the MXene nanosheets. For the Bi_2_MoO_6_/MXene-50%, the Bi_2_MoO_6_ nanoplates are sparsely wrapped with the MXene nanosheets (Fig. 2c, d). When the Bi_2_MoO_6_/MXene mass ratio increases to 70:30, the uniform and dense distribution of Bi_2_MoO_6_ is observed, almost covering the entire surface of the MXene nanosheets (Fig. 2e, f), implying an optimized mass ratio. As the Bi_2_MoO_6_ content continues to rise to 90 wt%, the Bi_2_MoO_6_ nanoplates evidently aggregate, as the insufficient surface area of the MXene nanosheets cannot load and anchor so many Bi_2_MoO_6_ nanoplates (Fig. 2g, h). TEM images were employed to further observe the structures of the Bi_2_MoO_6_/MXene heterostructures. It can be seen that the Bi_2_MoO_6_ nanoplates anchored on the MXene nanosheets have an irregular structure. The Bi_2_MoO_6_/MXene-50% (Fig. 3a, b), Bi_2_MoO_6_/MXene-30% (Fig. 3c, d), and Bi_2_MoO_6_/MXene-10% (Fig. 3e, f) display sparse, moderate, and dense distribution of Bi_2_MoO_6_ on the MXene nanosheets, respectively, in accordance with the corresponding SEM images. The STEM and corresponding element mappings (Fig. 3f) reveal similar images of Bi, Mo, Ti, and C elements, demonstrating the homogeneous distribution of Bi_2_MoO_6_ in the composite Bi_2_MoO_6_/MXene-30%.

**Fig. 4:**
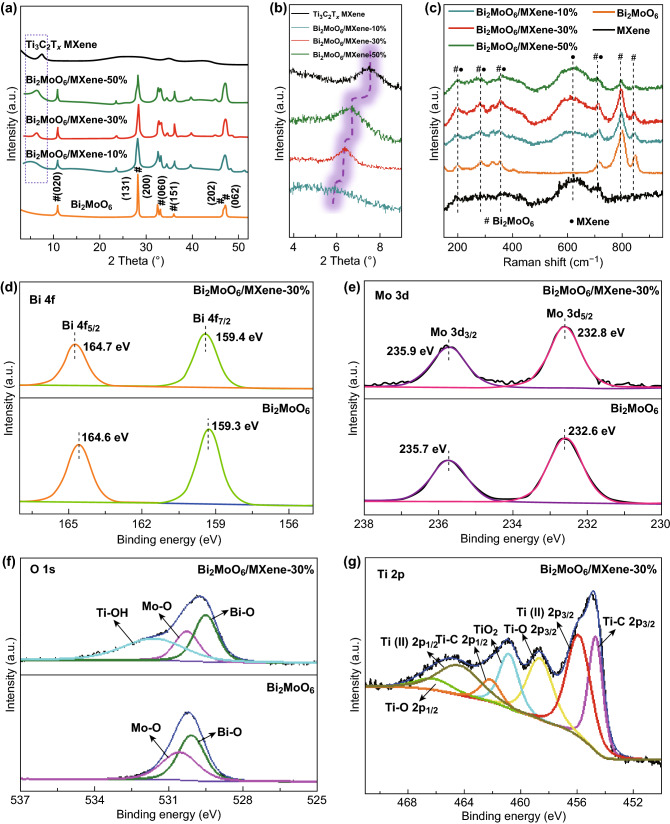
Characterization of Bi_2_MoO_6_/MXene heterostructures: **a**, **b** XRD patterns, **c** Raman speatra of the Bi_2_MoO_6_/MXene heterostructures. High-resolution of **d** Bi 4*f*, **e** Mo 3*d*, **f** O 1*s*, and **g** Ti 2*p* XPS spectrum of Bi_2_MoO_6_/MXene-30%

XRD analysis was conducted to identify the composition of the samples, as shown in Fig. 4a. The XRD pattern of the as-prepared Bi_2_MoO_6_ nanoplates consists of strong (020) peak at 10.9° and (131) peak at 28.2° as well as several weak peaks corresponding to the (200), (060), (151), (202), and (062) planes of pristine Bi_2_MoO_6_ [49–51]. After the assembly process, the (002) peak of MXene occurs in the patterns of the composites, indicating the successful combination of Bi_2_MoO_6_ and MXene. Moreover, Fig. 4b displays the regional XRD profiles of the Bi_2_MoO_6_/MXene-10%, Bi_2_MoO_6_/MXene-30%, Bi_2_MoO_6_/MXene-50%, and pristine MXene, which is marked by a purple frame in Fig. 4a. The (002) peak of MXene downshifts as the mass ratio of the MXene decreases in the composites, which is attributed to the gradually increased lattice spacing of MXene expanded by the Bi_2_MoO_6_ nanoplates. The structures of the Bi_2_MoO_6_/MXene heterostructures were also characterized by Raman spectroscopy, and the results are shown in Fig. 4c. The pristine Bi_2_MoO_6_ displays distinct phonon modes in the range of 200–1000 cm^−1^, signifying the vibrational modes of orthorhombic Bi_2_MoO_6_. Specifically, the bands located at 202, 285, 324, 355, and 404 cm^−1^ can be ascribed to the bending, wagging, and twisting modes of Mo–O bonds, while the bands at 713, 796, and 848 cm^−1^ correspond to the stretching modes of Mo–O bond [28, 31]. Notably, besides the band situated at 621 cm^−1^ (*ω*_4_) corresponding to *E*_*g*_ in-plane vibration of surface functional group atoms, the bands of the pristine MXene show similar locations to the Bi_2_MoO_6_, including the bands located at 193 cm^−1^ (*ω*_2_) and 711 cm^−1^ (*ω*_3_) for A 1 g symmetry out-plane vibrations of Ti and C atoms and those at 284 cm^−1^ (*ω*_5_) and 356 cm^−1^ (*ω*_5_) for *E*g in-plane vibration of Ti and C atoms [52, 53]. The Bi_2_MoO_6_/MXene heterostructures display all the characteristic peaks belonging to Bi_2_MoO_6_ (e.g., 713, 796, and 848 cm^−1^) and MXene (621 cm^−1^), and the increase in MXene content in the heterostructures leads to the stronger peaks for MXene together with the weaker peaks for Bi_2_MoO_6_.

XPS was carried out to evaluate the surface chemical properties of the Bi_2_MoO_6_ nanoplates, pristine MXene, and Bi_2_MoO_6_/MXene-30%. The XPS survey of the Bi_2_MoO_6_/MXene-30% comprises the characteristic peaks of Bi_2_MoO_6_ and MXene, such as Ti 2*p* peak for Ti_3_C_2_T_*x*_ as well as Bi 4*f* and Mo 3*d* peaks for Bi_2_MoO_6_ (Fig. S6), confirming the strong integration between the Bi_2_MoO_6_ nanoplates and the MXene nanosheets. High-resolution XPS spectra of Bi 4*f* and Mo 3*d* core levels of Bi_2_MoO_6_/MXene-30% and Bi_2_MoO_6_ are shown in Fig. 4d, e. The Bi 4*f* core level of Bi_2_MoO_6_/MXene-30% displays two peaks at 159.4 and 164.7 eV, related to Bi 4*f*_7/2_ and Bi 4*f*_5/2_ of the Bi^3+^, respectively. Meanwhile, the Mo 3*d* core level of Bi_2_MoO_6_/MXene-30% could be divided into two peaks situated at 232.8 and 235.9 eV, which can be assigned to Mo 3*d*_5/2_ and Mo 3*d*_3/2_ of Mo^6+^, respectively. It is noteworthy that the peaks of Bi 4*f* and Mo 3*d* core levels of Bi_2_MoO_6_/MXene-30% have higher binding energies than those of the Bi_2_MoO_6_, implying that the Bi_2_MoO_6_ nanoplates become more electrochemically active with the assistance of MXene. As shown in Fig. 4f, the O 1*s* core level of Bi_2_MoO_6_ is fitted with two components centered at 529.5 and 530.3 eV, which could be ascribed to Bi–O and Mo–O bonds, respectively [30, 47, 48]. Different from the Bi_2_MoO_6_, the Bi_2_MoO_6_/MXene-30% shows an additional peak at 531.6 eV corresponding to Ti–OH bond due to the presence of the MXene. Ti 2*p* XPS spectra are also showed in Fig. 4g to identify the chemical composition of the MXene. The Ti 2*p* core level was fitted with seven components, including three doublets (Ti 2*p*_3/2_–Ti 2*p*_1/2_) and a single peak located at 460.8 eV. The three doublets centered at 454.7/462.2, 455.9/464.4, and 458.7/466.1 eV could be assigned to Ti–C, Ti(II), and Ti–O, respectively, while the single peak might result from TiO_2_, implying the partial oxidation of the MXene during the construction of the Bi_2_MoO_6_/MXene heterostructure [40, 50].

**Fig. 5:**
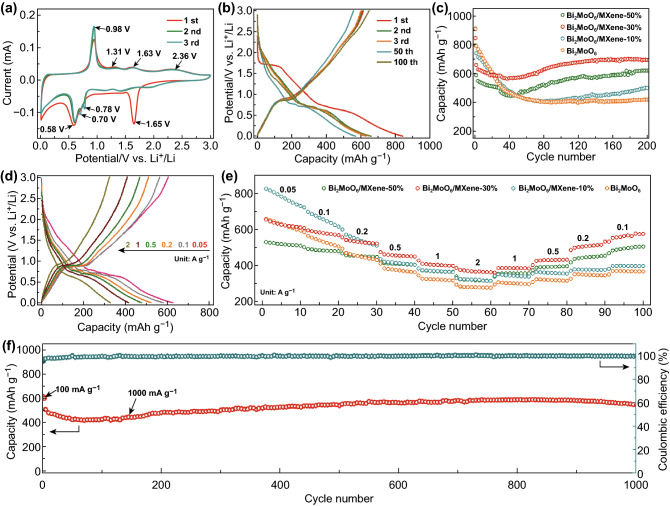
Electrochemical performance of the Bi_2_MoO_6_/MXene electrodes: **a** CV curves of Bi_2_MoO_6_/MXene-30% for the first three cycles at 0.1 mV s^−1^. **b** Charge/discharge profiles of Bi_2_MoO_6_/MXene-30% at 100 mA g^−1^ at different cycles. **c** Cycling performance of Bi_2_MoO_6_/MXene-50%, Bi_2_MoO_6_/MXene-30%, Bi_2_MoO_6_/MXene-10%, and pristine Bi_2_MoO_6_ electrodes at 100 mA g^−1^ for 200 cycles. **d** Charge/discharge profiles of Bi_2_MoO_6_/MXene-30% at different current rates. **e** Comparison of rate capabilities of Bi_2_MoO_6_/MXene-30% at various current rates from 50 to 2000 mA g^−1^. **f** Long-term cycling performance of Bi_2_MoO_6_/MXene-30% in 1000 cycles at 1 A g^−1^

The lithium-ion storage behaviors of the Bi_2_MoO_6_/MXene composite electrodes and the Bi_2_MoO_6_ electrode were first explored by cyclic voltammetry (CV) for the first three cycles at a scan rate of 0.1 mV s^−1^ within a voltage range of 0.01–3 V, as shown in Fig. 5a and S7. All the electrodes show irreversible peaks at around 1.65, 1.26, and 0.58 V in the first cathodic scan, which could be attributed to the insertion of Li^+^ into layer structure of the Bi_2_MoO_6_ crystal, the irreversible transformation from Bi_2_MoO_6_ to Bi and Mo metal (Eq. 2), the electrolyte decomposition, and formation of solid electrolyte interface (SEI) coupled with the alloying transformation from Bi to Li_3_Bi, respectively [29–31]. Especially, the board peak at 0.58 V was subsequently split into several minor peaks, corresponding to the lithiation process of Bi to LiBi (Eq. 3) and Li_3_Bi (Eq. 4). In the anodic scan, a strong peak at 0.98 V was observed which is caused by the de-alloying reaction of Li_3_Bi. Besides, several board peaks at 1.31, 1.63, and 2.36 V are related to the oxidation of Mo (Eq. 5) and Bi (Eq. 6), respectively [28, 31]. The electrochemical reactions of the Bi_2_MoO_6_ are listed as follows:

2 $${\text{Bi}}_{2} {\text{MoO}}_{6} + 12{\text{Li}}^{ + } + 12e^{ - } \to 2{\text{Bi}} + {\text{Mo}} + 6{\text{Li}}_{2} {\text{O}}$$

3 $${\text{Bi}} + {\text{Li}}^{ + } + e^{ - } \leftrightarrow {\text{LiBi}}$$

4 $${\text{LiBi}} + 2{\text{Li}}^{ + } + 3e^{ - } \leftrightarrow {\text{Li}}_{3} {\text{Bi}}$$

5 $$2{\text{Bi}} + 3{\text{Li}}_{2} {\text{O}} \leftrightarrow {\text{Bi}}_{2} {\text{O}}_{3} + 6{\text{Li}}^{ + } + 6e^{ - }$$

6 $${\text{Mo}} + x{\text{Li}}_{2} {\text{O}} \leftrightarrow {\text{MoO}}_{x} + 2x{\text{Li}}^{ + } + 2xe^{ - } \quad \left( {x = 2,3} \right)$$

The galvanostatic charge/discharge curves of the electrodes were conducted at a current density of 100 mA g^−1^ in the voltage range of 0.01–3 V (Figs. 5b and S8), which show the electrochemical behaviors in accordance with the CV curves. The initial charge and discharge capacity of Bi_2_MoO_6_/MXene-30% is 615.5 and 844.2 mAh g^−1^, respectively, leading to an initial coulombic efficiency (ICE) of 72.9%. The capacity loss in the first cycle might be attributed to the formation of SEI layer coupled with the irreversible reactions as mentioned above [31, 54, 55]. The ICE of Bi_2_MoO_6_/MXene-30% (72.9%) is lower than those of Bi_2_MoO_6_/MXene-10% (76.6%) and Bi_2_MoO_6_ (82.6%), but higher than that of Bi_2_MoO_6_/MXene-50% (71.2%). The possible reason for this phenomenon is that with higher MXene content, the increased surface area of MXene results in more irreversible reactions between Li^+^ and the functional groups on the MXene surface, such as –O, –F, and –OH [47, 56].

The comparison of the cycling performance of the Bi_2_MoO_6_/MXene composite electrodes and the Bi_2_MoO_6_ electrode is given in Fig. 5c at a current density of 100 mA g^−1^. The Bi_2_MoO_6_/MXene-30% delivers a stable capacity of 692 mAh g^−1^ after 200 cycles, much higher than those of Bi_2_MoO_6_/MXene-50% (617.5 mAh g^−1^), Bi_2_MoO_6_/MXene-10% (497.6 mAh g^−1^), and the pristine Bi_2_MoO_6_ (416.1 mAh g^−1^). It indicates that the optimized ratio of the composite is beneficial for maximizing the lithium storage capacity of the Bi_2_MoO_6_/MXene. Furthermore, a capacity fading occurs in the initial dozens of cycles followed by a capacity reactivation process and an eventual capacity stabilization. The capacity fading results from the mechanical degradation of the electrode structure and the formation of an unstable SEI layer, while the subsequent capacity reactivation and stabilization could be ascribed to a restructuring process as well as stable SEI formation with cycling [57].

Figures 5d and S9 show the charge/discharge curves of Bi_2_MoO_6_/MXene-30% and the pristine Bi_2_MoO_6_ at various current densities from 0.05 to 2 A g^−1^. As seen, the pristine Bi_2_MoO_6_ has a slightly higher capacity of 629.4 mAh g^−1^ at the small current density while it could only deliver capacities of 375.6, 323.7, and 279.8 mAh g^−1^ at 0.5, 1, and 2 A g^−1^, respectively. By contrast, the specific capacity of Bi_2_MoO_6_/MXene-30% is measured to be 626.5, 580.6, 522.1, 477, 414.8, and 328.2 mAh g^−1^ at 0.05, 0.1, 0.2, 0.5, 1, and 2 A g^−1^, respectively. It demonstrates that the conductive MXene effectively enhances the rate performance of the composite electrode. As displayed in Fig. 5e, the Bi_2_MoO_6_/MXene-30% exhibits the best rate capability from 0.05 to 2 A g^−1^ compared with the Bi_2_MoO_6_/MXene-50%, Bi_2_MoO_6_/MXene-10%, and Bi_2_MoO_6_ electrodes. When the current density is turned back from 2 to 0.1 A g^−1^, the specific capacity of Bi_2_MoO_6_/MXene-30% are recovered up to 566.3 mAh g^−1^, signifying the best reversibility and structural stability compared with other electrodes. Furthermore, to estimate the long-term cycling stability, Bi_2_MoO_6_/MXene-30% was charged/discharged for 1000 cycles at a high current density of 1 A g^−1^ (Fig. 5f). After the first three cycles at 0.1 A g^−1^ for activation, the Bi_2_MoO_6_/MXene-30% exhibits a reversible capacity of 507.2 mAh g^−1^ at the fifth cycle and retains a capacity of 545.1 mAh g^−1^ with 99.6% coulombic efficiency after 1000 cycles. The delivered capacity of Bi_2_MoO_6_/MXene-30% gradually increases with cycling, which is caused by the restructuring process and the subsequent formation of a stable structure during repeated lithiation/delithiation [57]. The SEM images of Bi_2_MoO_6_/MXene-30% before and after 1000 cycles at 1 A g^−1^ were explored to show the structural stability of the electrode as displayed in Fig. S10. It could be clearly seen that the Bi_2_MoO_6_ was wrapped by the MXene nanosheets, which are consistent with the SEM images of the Bi_2_MoO_6_/MXene-30% materials (Fig. 2e, f). After 1000 charge/discharge cycles, the surface of the electrode turns rough, but no obvious crack was observed, indicating the excellent structural stability of the Bi_2_MoO_6_/MXene-30% electrode during lithiation/delithiation.

**Fig. 6:**
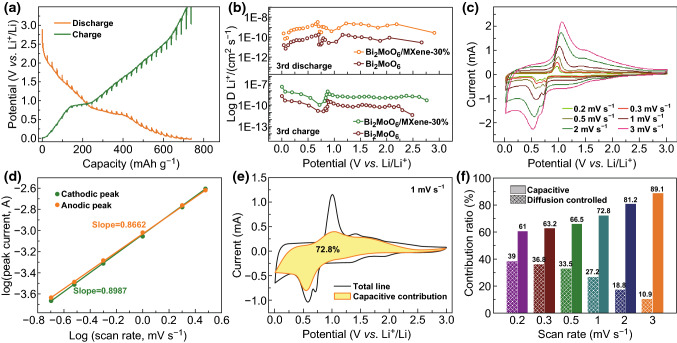
Electrochemical kinetic analysis of Li storage behavior of Bi_2_MoO_6_/MXene-30%. **a** GITT profiles (current pulse at 100 mA g^−1^ for 30 min followed by 1 h relaxation), **b** diffusion coefficients calculated from GITT profiles according to overpotential, **c** CV curves at various scan rates from 0.2 to 3 mV s^−1^ in the voltage range of 0.01–3 V (vs. Li^+^/Li), **d** log(*i*)–log(*v*) curves, **e** CV profile measured at 1 mV s^−1^ with shaded area displaying the pseudocapacitive contribution, and **f** normalized proportions of capacitive and diffusion-controlled contribution at various scan rates

To study the origins of the better rate capability of Bi_2_MoO_6_/MXene-30%, the lithium diffusion coefficients of Bi_2_MoO_6_/MXene-30% and Bi_2_MoO_6_ were calculated by GITT according to Eq. 1, as shown in Figs. 6a, b and S10. Compared with the Bi_2_MoO_6_, Bi_2_MoO_6_/MXene-30% exhibits lower overpotential and higher diffusion coefficients during the lithiation/delithiation process, implying better reaction kinetics [58, 59]. The enhanced reaction kinetics of Bi_2_MoO_6_/MXene-30% can be associated with the decoration of the highly conductive MXene nanosheets, which support and contact with the Bi_2_MoO_6_ nanoplates sufficiently to efficiently improve the charge transport. Additionally, the EIS spectra show that the Bi_2_MoO_6_/MXene-30% has the lowest *R*_ct_ resistance of 105.9 Ω and *R*_s_ resistance of 3.08 Ω among these four samples, verifying its enhanced reaction kinetics for lithium-ion storage (Fig. S12a, b, Table S1). The impedance behaviors of the Bi_2_MoO_6_/MXene-30% and Bi_2_MoO_6_ electrodes were explored by the complex model of capacity to confirm the promotion of MXene for rapid diffusion and transportation of lithium ions (Fig. S12c, d). After the decoration of MXene, the Bi_2_MoO_6_/MXene-30% displays much lower minimal characteristic relaxation time constant τ_0_ (919 ms) and downtrend of normalized *C*′(ω) than the pristine Bi_2_MoO_6_ electrodes, implying faster transport and diffusion of electrolyte ions in the Bi_2_MoO_6_/MXene-30% electrode [52].

CV measurements were taken to unravel the charge storage kinetics of Bi_2_MoO_6_/MXene-30%. Figure 6a depicts the CV curves of Bi_2_MoO_6_/MXene-30% at various scan rates from 0.2 to 3 mV s^−1^. The charge storage mechanism could be analyzed by the following formula, which shows the relationship between the measured current (*i*) and the scan rate (*v*):

7 $$i = av^{b}$$

where *b* could be calculated from the slope of the fitted log(*i*) − log(*v*) curves [60, 61], distinguishing the electrochemical behavior as a diffusion-controlled process (*b* = 0.5) or a non-diffusion-controlled behavior (*b* = 1). As shown in Fig. 6d, the *b* value of Bi_2_MoO_6_/MXene-30% is 0.8987 at 0.58 V in the cathodic scan and 0.8662 at 0.97 V in the anodic scan, indicating a fast charge storage kinetic dominated by pseudocapacitive behavior. This behavior can also be confirmed by quantifying the pseudocapacitive contribution based on Eq. 8:

8 $$i\left( v \right) = k_{1} v + k_{2} v^{1/2}$$

where *i*(*v*), *k*_1_*v*, *k*_2_*v*^1/2^, and *v* are the measured current at a certain potential, the capacitive-dominated current, the diffusion-controlled current, and the corresponding scan rate, respectively. The capacitive-dominated current at a certain scan rate could be obtained via calculating the value of *k*_1_ [60, 61]. Figures 6e and S13 depict the CV curves of Bi_2_MoO_6_/MXene-30% at various scan rates, in which the shaded portion stands for the capacitive-dominated region, while the non-shaped portion means the diffusion-controlled region. It can be seen that up to 72.8% of the charge is contributed by the pseudocapacitive behavior at 1 mV s^−1^. Moreover, the pseudocapacitive contribution of the Bi_2_MoO_6_/MXene-30% electrode enhances with the scan rate increasing (Fig. 6f). The capacitive-dominated mechanism coupled with the highly conductive MXene can offer ultrafast lithium-ion storage, endowing Bi_2_MoO_6_/MXene-30% with enhanced rate capability and cycling stability.

The above results suggest that the lithium storage property of Bi_2_MoO_6_ could be effectively enhanced by introducing highly conductive MXene as a substrate to fabricate a plate-to-layer Bi_2_MoO_6_/MXene heterostructure. In the heterostructure, the MXene nanosheets can promote the charge transport and alleviate the volume change of the Bi_2_MoO_6_, leading to high specific capacity, superior rate capability, and excellent long-term cycling stability. The Bi_2_MoO_6_/MXene-30% exhibits competitive performance compared with other TMOs-based electrodes, indicating its promising potential as an anode material of LIBs.

## Conclusions

To solve the problems of Bi_2_MoO_6_ as an electrode material for LIBs, i.e., low electronic conductivity and huge volume change, we have fabricated Bi_2_MoO_6_ nanoplates on highly conductive Ti_3_C_2_T_*x*_ MXene nanosheets to form a plate-to-layer heterostructure via a simple electrostatic self-assembled method. In the Bi_2_MoO_6_/MXene heterostructure, the MXene nanosheets cannot only promote the electron transfer and facilitate the Li^+^ transport, but also accommodate the volume expansion/contraction of Bi_2_MoO_6_ during lithiation/delithiation, endowing the composite electrodes with high conductivity, good structural stability, and excellent electrochemical durability. As a result, the Bi_2_MoO_6_/MXene-30% exhibits remarkably enhanced lithium storage properties, presenting a specific capacity of 692 mAh g^−1^ at 100 mA g^−1^ after 200 cycles, a superior rate capability of 328.2 mAh g^−1^ at 2 A g^−1^ as well as an outstanding cycling durability with a capacity of 545.1 mAh g^−1^ and 99.6% coulombic efficiency at 1 A g^−1^ after 1000 cycles. The Bi_2_MoO_6_/MXene heterostructure with competitive performance is conceivable to be a promising high-performance anode material for LIBs. Furthermore, various TMOs/Ti_3_C_2_T_*x*_ composites were suggested to be explored using Ti_3_C_2_T_*x*_ MXene nanosheets as a conductive substrate to achieve good electrochemical performance and application in energy storage.

## Electronic supplementary material

Below is the link to the electronic supplementary material.

Supplementary material 1 (PDF 897 kb)
