# Supplementary material for: Plate-to-Layer Bi2MoO6/MXene-Heterostructured Anode for Lithium-Ion Batteries
Source: Nanomicro Lett. 2019 Sep 25;11:81. doi: 10.1007/s40820-019-0312-y (PMC7770671; doi:10.1007/s40820-019-0312-y)
Supplement: Supplementary file 1 — Supplementary material 1 (PDF 897 kb) [file 40820_2019_312_MOESM1_ESM.pdf]

Supporting Information for

Plate-to-layer Bi_2MoO_6/MXene Heterostructured Anode for Lithium Ion Batteries

Peng Zhang[1], Danjun Wang[1, 2], Qizhen Zhu[1, *], Ning Sun[1], Feng Fu[2, *], Bin Xu[1, *]

[1]State Key Laboratory of Organic-Inorganic Composites, Beijing Key Laboratory of Electrochemical Process and Technology for Materials, Beijing University of Chemical Technology, Beijing 100029, People's Republic of China

[2]Shaanxi Key Laboratory of Chemical Reaction Engineering, School of Chemistry and Chemical Engineering, Yan'an University, Yan'an 716000, People's Republic of China

*Corresponding authors. E-mail: binxumail@163.com (B. Xu), Zhuqz@mail.buct.edu.cn (Q. Zhu), yadxfufeng@126.com (F. Fu)

Supplementary Figures and Table

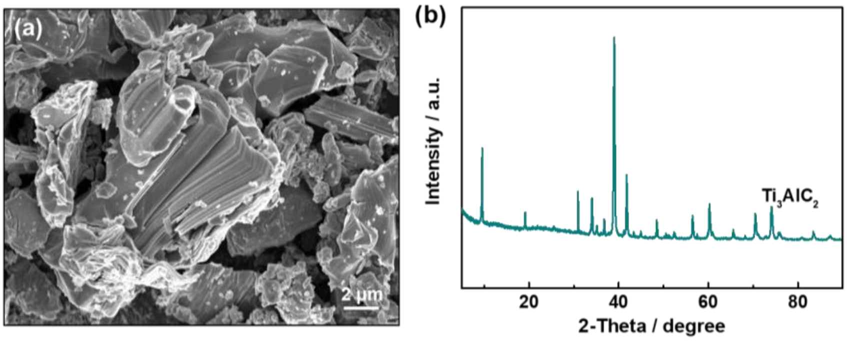

Fig. S1 a SEM image, **b** XRD pattern of the Ti_3AlC_2 MAX phase

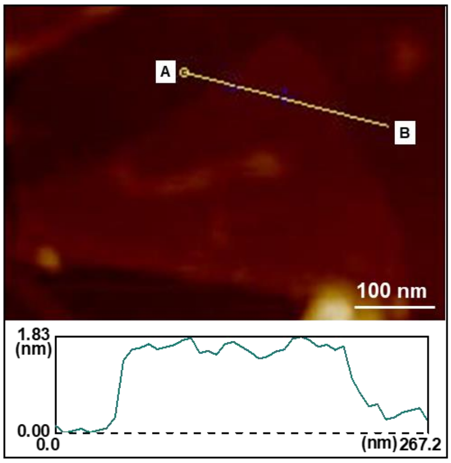

Fig. S2 AFM image of the pristine MXene nanosheets

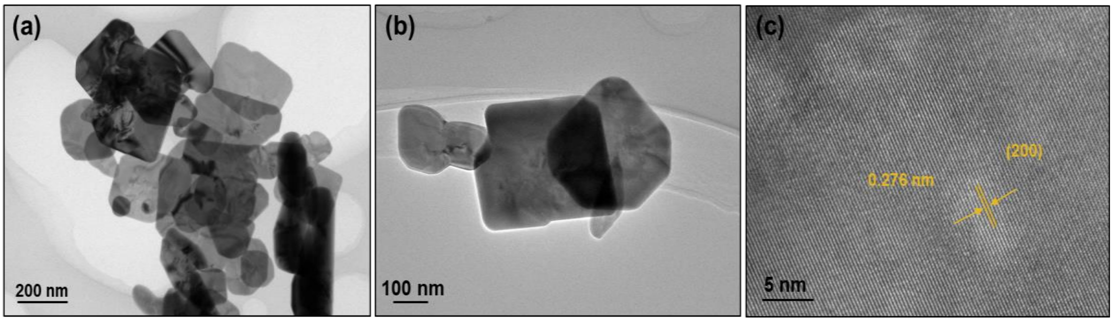

Fig. S3 a, b TEM images, **c** HRTEM image of the pristine Bi_2MoO_6 nanoplates

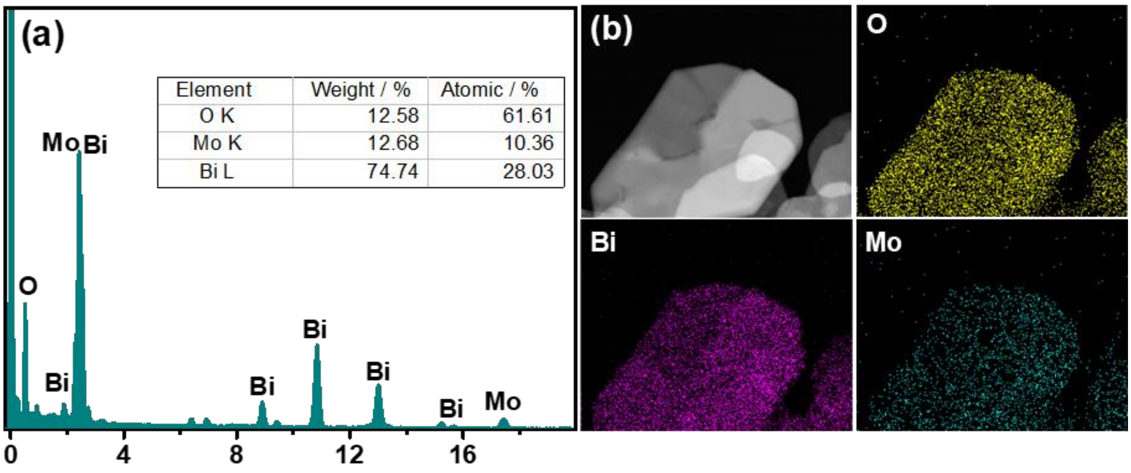

Fig. S4 a EDS and **b** corresponding elemental mapping images of the Bi_2MoO_6 nanoplates

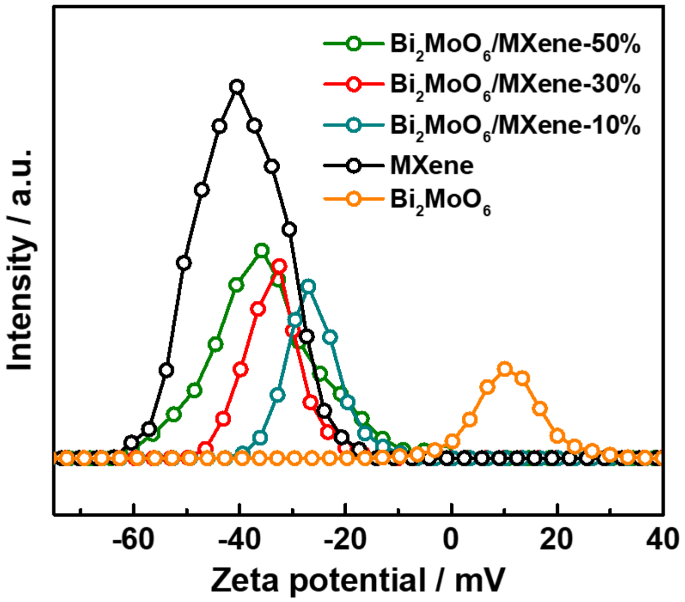

Fig. S5 Zeta potentials of the as-prepared samples

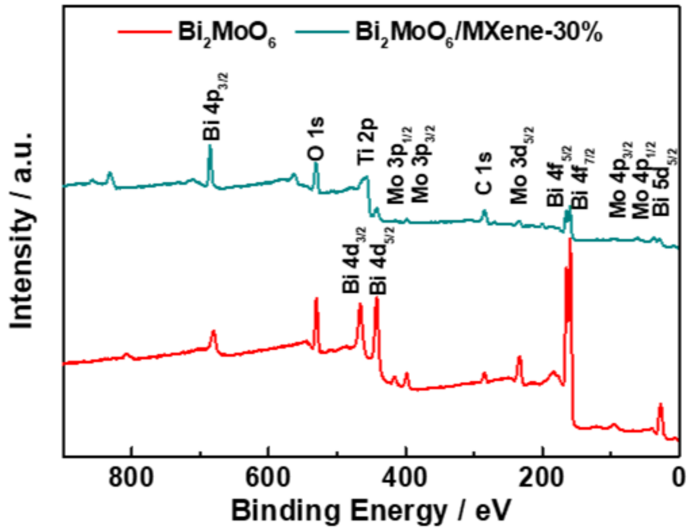

Fig. S6 XPS survey of Bi_2MoO_6/MXene-30% and Bi_2MoO_6

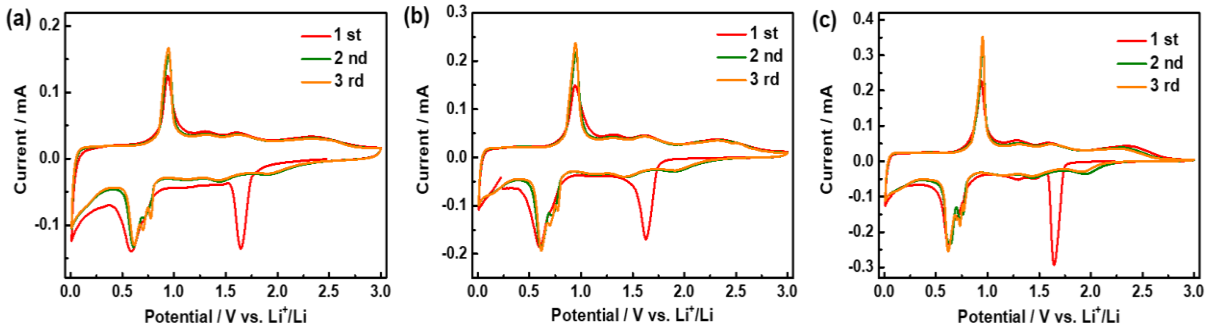

Fig. S7 CV curves of **a** Bi_2MoO_6/MXene-50%, **b** Bi_2MoO_6/MXene-10%, and **c** Bi_2MoO_6

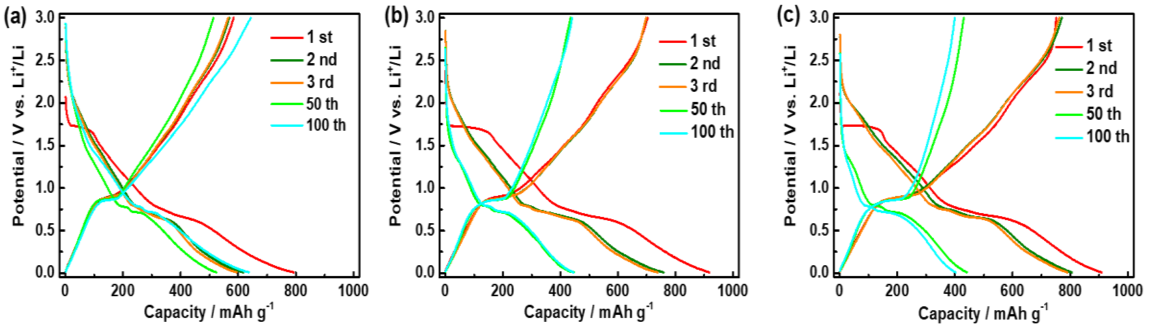

Fig. S8 Charge/discharge profiles of **a** Bi_2MoO_6/MXene-50%, **b** Bi_2MoO_6/MXene-10%, and **c** Bi_2MoO_6

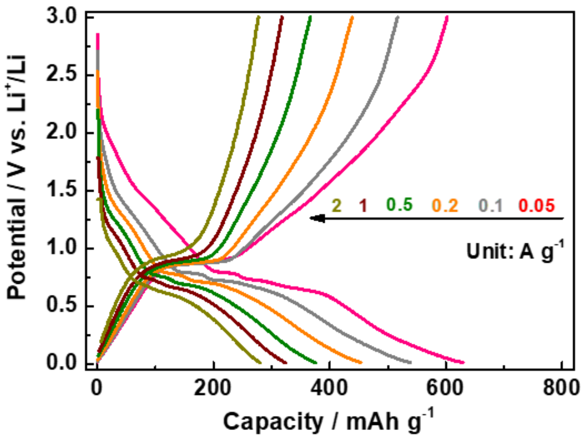

Fig. S9 Charge/discharge profiles of the Bi_2MoO_6 electrode at different current rates

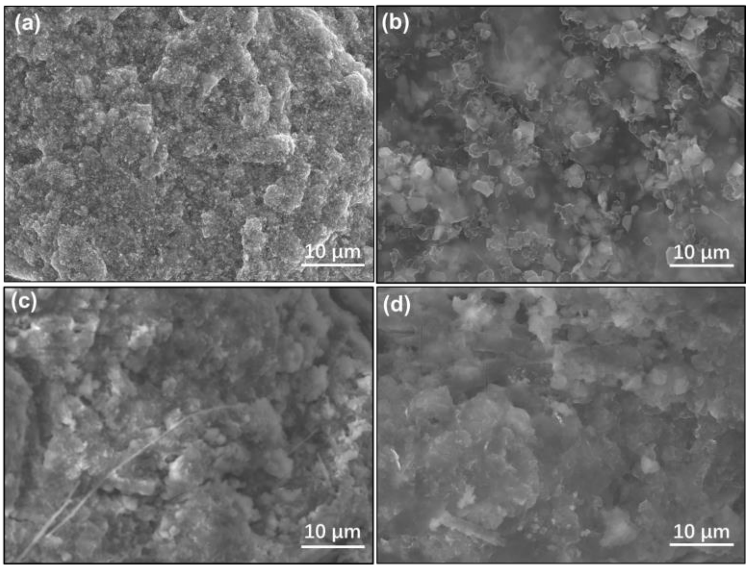

Fig. S10 SEM images of the Bi_2MoO_6/MXene-30% electrode: **a, b** before cycling; **c, d** after 1000 cycles at 1 A g^{-1}

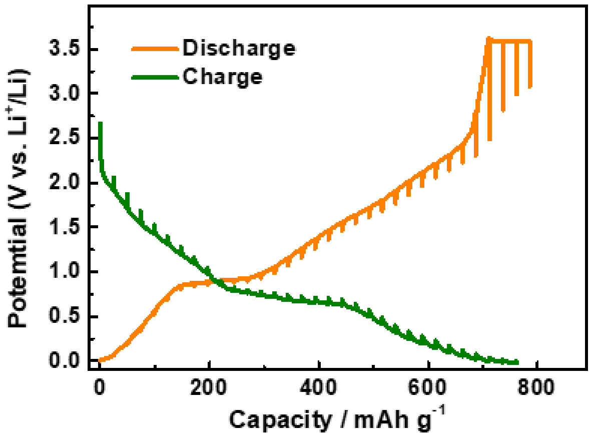

Fig. S11 GITT profiles of the Bi_2MoO_6 (current pulse at 100 mA g^{-1} for 30 min followed by 1 h relaxation)

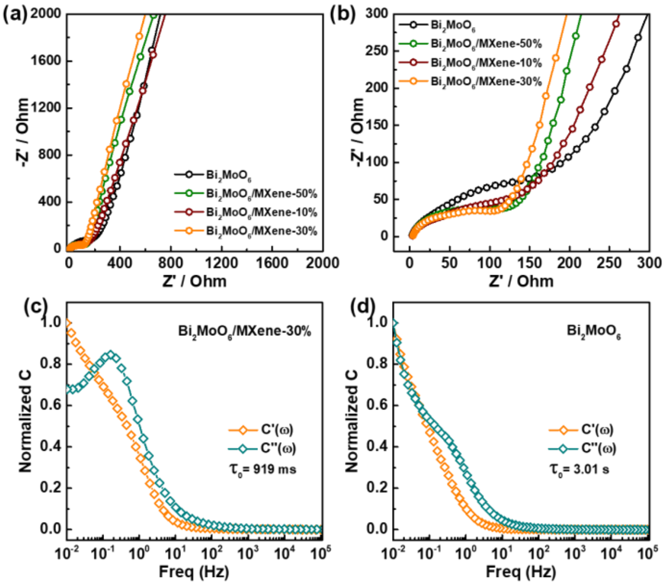

Fig. S12 a, b EIS spectra of the Bi_2MoO_6/MXene-50%, Bi_2MoO_6/MXene-30%, Bi_2MoO_6/MXene-10%, and Bi_2MoO_6 electrodes. Normalized real and imaginary capacities of the **c** Bi_2MoO_6/MXene-30% and **d** Bi_2MoO_6 electrodes

The real C′(ω) and imaginary C″(ω) capacities could be calculated through Eqs. 1 and 2:

$$C'(\omega) = \frac{-Z''(\omega)}{\omega|Z(\omega)|^2} \quad (1)$$

$$C''(\omega) = \frac{Z''(\omega)}{\omega|Z(\omega)|^2} \quad (2)$$

where $Z'(\omega)$ and $Z''(\omega)$ are the real and imaginary parts of the complex impedance Z (ω), respectively. The angular frequency (ω) is calculated by $\omega = 2\pi f$.

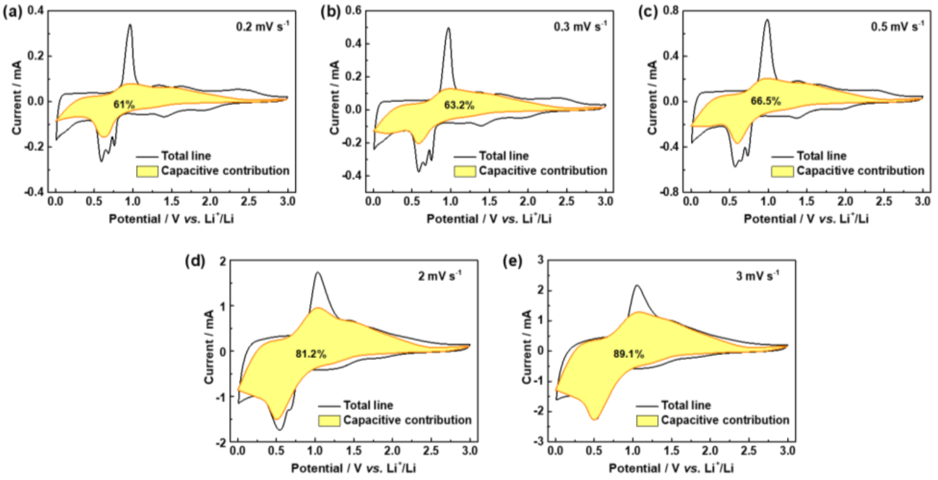

Fig. S13 CV profile measured at **a** 0.2 mV s^{-1}, **b** 0.3 mV s^{-1}, **c** 0.5 mV s^{-1}, **d** 2 mV s^{-1}, and **e** 3 mV s^{-1} with shaped area displaying the capacitive contribution

Table S1 Fitting EIS data of the as-prepared samples

	Bi_2MoO_6/MXene-50%	Bi_2MoO_6/MXene-30%	Bi_2MoO_6/MXene-10%	Bi_2MoO_6
R_s/Ω	3.49	3.08	3.76	4.18
R_{ct}/Ω	115.8	105.9	121.0	148.9
